# Reasons for discontinuation of subcutaneous interferon β-1a three times a week among patients with multiple sclerosis: a real-world cohort study

**DOI:** 10.1186/s12883-017-0831-4

**Published:** 2017-03-23

**Authors:** Meritxell Sabidó-Espin, Rick Munschauer

**Affiliations:** 1 Frankfurter Str. 250, HPC: F135/201, Darmstadt, 64293 Germany; 2 EMD Serono, Rockland, MA USA

**Keywords:** Interferon β-1a, Multiple sclerosis, Adherence, Discontinuation

## Abstract

**Background:**

Continuation of interferon (IFN) β-based therapies is important for maximum treatment effectiveness in patients with multiple sclerosis (MS); however, few real-world data are available on discontinuation from IFN β. The aim of this cohort analysis was to estimate real-world discontinuation rates up to 3 years among MS patients in the United States taking subcutaneous (sc) IFN β-1a three times a week (tiw) and to identify whether the factors associated with discontinuation change over time.

**Methods:**

Patient data were pooled from the MarketScan^©^ Commercial and Medicare Supplemental healthcare claims databases. Patients with ≥1 multiple sclerosis diagnosis who were sc IFN β-1a tiw naïve, had ≥1 year of continuous eligibility before treatment, and ≥1 prescription were followed from first prescription (index date) until date of discontinuation, switch, or end of observation. Treatment status was analysed at exactly 1, 2 or 3 years after index. Multivariable models were used to identify drivers of discontinuation.

**Results:**

Data from 5956 patients were included; 2862 patients (48.1%) discontinued therapy. Discontinuation rates were 36.9% (1 year), 49.5% (2 years) and 55.8% (3 years). A greater proportion of discontinuing patients had poor adherence (<80% [94.0%] versus ≥80% [51.7%]) or were taking additional medication at follow-up versus the overall population. Factors independently associated with discontinuation irrespective of time on therapy were increasing number of magnetic resonance imaging scans (1 year adjusted odds ratio 1.45, 95% confidence interval 1.26–1.67; 2 years 1.18, 1.06–1.32; 3 years 1.20, 1.07–1.34) and adherence <80% versus ≥80% (1 year 180.95, 135.84–241.03; 2 years 135.80, 100.10–184.23; 3 years 174.89, 115.27–265.38). Factors associated only with early discontinuation (at 1 year) were ≥3 sets of laboratory investigations versus none (2.54, 1.20–5.38), and anxiolytic use at follow-up (1.40, 1.06–1.82). Factors associated only with later discontinuation (at 2 years and/or at 3 years) were antidepressant use at follow-up (2 years 1.46, 1.10–1.94) and greater number of relapses (2 years 1.60, 1.11–2.30; 3 years 2.31, 1.27–4.22).

**Conclusions:**

Potential drivers of discontinuation change over time. Improved awareness of the drivers of discontinuation could lead to targeted interventions to improve adherence.

**Electronic supplementary material:**

The online version of this article (doi:10.1186/s12883-017-0831-4) contains supplementary material, which is available to authorized users.

## Background

The chronic nature of multiple sclerosis (MS) necessitates long-term treatment with a disease-modifying drug (DMD) to delay the progression of MS-related disability, reduce the frequency of relapses and prevent the formation of new brain lesions in patients with the relapsing-remitting form of the disease [1]. Adherence is defined as the ability and willingness to follow a prescribed treatment regimen correctly [2]. Good adherence to DMDs is essential, as it is associated with better clinical outcomes, such as reduced use of health care resources, lower costs, and improvements in patient quality of life [3–6].

Of the DMDs available for the treatment of MS, interferon (IFN) β-based therapies are some of the most widely prescribed [7]. Continued treatment with IFN-β therapy is important to achieve maximum treatment efficacy, [8] but data from clinical trials and registries show that IFN-β therapies have a treatment discontinuation rate of between 14 and 44%, which may lead to disease reactivation [9]. The causes of IFN-β discontinuation may also change as a function of time. A retrospective hospital-chart-based study recently showed a clear difference in stopping patterns of IFN-β therapy according to the length of time on treatment, with patients stopping IFN-β therapy due to side effects after a median of 13 months, while those discontinuing due to failure of therapy stopped after a median of 36 months [10].

The aim of this cohort analysis was to estimate IFN-β discontinuation rates among MS patients in the United States (US) receiving subcutaneous (sc) IFN β-1a three times a week (tiw), and to identify factors associated with stopping patterns according to time on treatment in a real-world setting, by using data from claims databases. This analysis has the potential to provide insights on potential strategies to improve medication-taking behaviour and help health care providers anticipate how the challenges associated with long-term therapy change over time.

## Methods

### Data sources

Data were pooled from two sources. The first was the longitudinal Truven MarketScan^©^ Commercial Claims and Encounters database, which contains claims for more than 138 million health plan members from the year 2000 onwards and is considered to be representative of the US commercially insured population. The second source was the Medicare Supplemental and Coordination of Benefits (Medicare) databases, which contain the pooled claims data of approximately 2.5 million claimers in the US annually who have Medicare Supplemental Insurance paid for by employers.

### Patients

Patient data were included in the retrospective analysis if patients were ≥18 years old on the year of the index date, had a diagnosis of MS (presence of ≥1 medical claim with a primary or secondary International Classification of Diseases, 9^th^ revision, Clinical Modification [ICD-9-CM] diagnosis code for MS [340]) and had initiated treatment with sc IFN β-1a tiw during the study period (January 1, 2007 to December 31, 2013), had no record of previous sc IFN β-1a tiw treatment recorded in the database for at least 1 year before the index date, had ≥1 pharmacy claim for sc IFN β-1a tiw after the index date (captured through National Drug Codes), and had ≥1 year of continuous eligibility of treatment initiation with sc IFN β-1a tiw. Patients with a prescription for sc IFN β-1a tiw without a recorded diagnosis code for MS were excluded, as were pregnant women. Patients were followed from first prescription for sc IFN β-1a tiw until therapy switch or discontinuation, end of insurance eligibility, or end of observation period, whichever occurred first.

Data were fully compliant with the Health Insurance Portability and Accountability Act of 1996 (HIPPA). Given that the study only involved de-identified data, Institutional Review Board review or approval was not required.

### Study measures

Patient demographics were captured at the time of the index prescription claim, with baseline characteristics based on the year preceding sc IFN β-1a tiw initiation (baseline period). Health care utilisation, adherence, persistence, sc IFN β-1a tiw treatment duration, and use of corticosteroids and other symptomatic therapies were measured during follow-up (≥1 prescription fill), including the index date.

Annualised relapse rates were calculated using a validated algorithm [11, 12] that defined an MS-related relapse as a claim in the primary position at any time during an in-patient hospitalisation, or a claim with an MS diagnosis code in the primary or secondary outpatient setting (including emergency room visits) in addition to a pharmacy or medical claim for a qualifying corticosteroid on the day of, or within 7 days after, the visit. Comorbidity burden was evaluated using the Charlson Comorbidity Index score [13].

Treatment adherence was operationalised as the number of days of medication supplied within a refill interval in relation to the number of days in the refill interval, also referred to as the medication possession ratio [14]. Treatment persistence was defined as the proportion of patients who continued on sc IFN β-1a tiw for a period of 1 year without a gap in therapy of ≥90 days, [15] and treatment duration was calculated as the time (in months) elapsed from index date to switch or complete discontinuation.

### Statistical analysis

All statistical analyses were performed using SAS 9.4 (SAS institute Inc., Cary, NC, USA). Frequency distributions for categorical variables and mean (standard deviation) or median (interquartile range [IQR]) for continuous variables were calculated.

Overall discontinuation (%) was measured using the total sample of patients and with variable follow-up time. Discontinuation at 1 year, 2 years, and 3 years was calculated using patients who were followed-up for at least 1, 2, and 3 years, respectively. The proportion of patients who discontinued, including those who switched to another drug after discontinuation, at 1 year, 2 years and 3 years was calculated for patients who were followed-up for at least 1, 2 and 3 years, respectively. Among patients who continued or discontinued, the mean (standard deviation [SD]) number of relapses per year and the proportion of patients with a high number of relapses was calculated for the patients who were followed-up for at least 1, 2 and 3 years respectively.

To identify potential factors associated with discontinuation, bivariate and multivariate logistic regression models were used, and results were expressed as odds ratios (OR) with 95% confidence interval (CI). Variables with an unadjusted OR at the 0.15 level were included in the initial multivariable model and a stepwise fitted procedure was used; a variable was retained in the model if the p value was <0.05. Kaplan-Meier curves were used to estimate time to discontinuation by relapsing activity for those with ≥2 sc IFN β-1a claims. The models were also run without adherence to examine the relationship of other independent variables with adherence.

## Results

### Patient characteristics

**Fig. 1:**
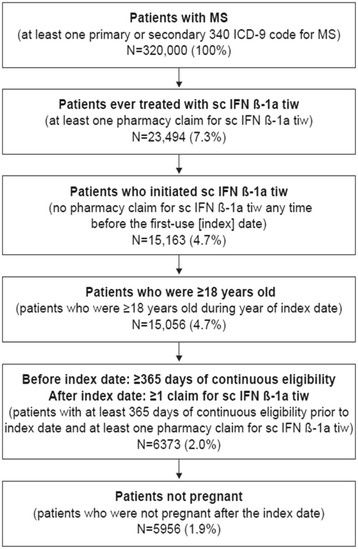
Patient flow selection. ICD-9, International Classification of Diseases, Revision 9. MS, multiple sclerosis. sc IFN β-1a tiw, subcutaneous interferon beta-1a three times a week. Numbers in brackets are the proportion of original patients

Overall, data from 5956 patients were included in this retrospective cohort analysis (Fig. 1). Baseline demographics and clinical characteristics are shown in Table 1. Most patients received specialist neurological care, rather than general or emergency medical care (Additional file 1: Table S1).

**Table 1:** Demographic and clinical characteristics of patients with multiple sclerosis initiating subcutaneous interferon β-1a, three times weekly, by discontinuation status

Characteristics	sc IFN β-1a tiw			
	Total sample (*N =* 5956)		Discontinued (*N =* 2862)	
	n	%	n	%
Female sex	4447	74.7	2177	76.1
Age, mean (SD) years	44 (10.7)		44 (10.8)	
Region				
Northeast	999	16.8	481	16.8
North Central	1764	29.6	766	26.8
South	2156	36.2	1066	37.2
West	968	16.3	517	18.1
Unknown	69	1.2	32	1.1
Charlson comorbidity index				
Index = 0	3974	66.7	1887	65.9
Index = 1	1102	18.5	552	19.3
Index = 2	501	8.4	242	8.5
Index ≥ 3	379	6.4	181	6.3
Relapse per year, mean (SD)	0.21 (0.57)		0.25 (0.55)	
High relapse (≥2 relapses)^a^	271	4.6	154	5.4
DMD use history	2162	36.3	1143	39.9
Treatment duration, median (IQR) months	9 (3–22)		6 (2–15)	
Persistence	5634	94.6	2645	92.4
Adherence to treatment <80% (vs. ≥80%)	3078	51.7	2691	94.0
Baseline corticosteroid use	2384	40.0	1194	41.7
Follow-up				
NSAID use	3221	54.1	1862	65.1
Antidepressant use	3020	50.7	1682	58.8
Anxiolytic use	1229	20.6	753	26.3
Corticosteroid use	2222	37.3	1362	47.6

*DMD* disease-modifying drug, *NSAID* non-steroidal anti-inflammatory drugs, *IFN* interferon, *IQR*, interquartile range, *SC* subcutaneous, *SD* standard deviation, Tiw three times a week

^a^High relapse activity defined as having ≥2 relapses in the first year prior to start of subcutaneous interferon β-1a, three times weekly

### Discontinuation of sc IFN β-1a tiw

In total, 2862 patients (48.1%) discontinued sc IFN β-1a tiw; the median treatment duration was 6 months (Table 1). The clinical characteristics of the patients who discontinued were similar to those reported for the total sample, although a greater proportion of discontinuing patients presented with low adherence (adherence <80%, 94.0% versus 51.7%, respectively). In addition, a greater proportion were taking additional medication at follow-up versus the overall population (non-steroidal anti-inflammatory drugs, 65.1% versus 54.1%; antidepressants, 58.8% versus 50.7%; anxiolytics, 26.3% versus 20.6%; corticosteroids, 47.6% versus 37.3%) (Table 1).

The discontinuation rates at 1, 2 and 3 years were 36.9% (1470 of 3975 patients)), 49.5% (1282 of 2592 patients), and 55.8% (928 of 1664 patients), respectively. Among those who discontinued, 20.6% did not switch to another drug at 1 year, 22.9% at 2 years, and 23.9% at 3 years. The proportion who discontinued and switched to another drug was 16.4% at 1 year, 26.6% at 2 years, and 31.9% at 3 years.

### Factors associated with sc IFN β-1a tiw discontinuation

Factors independently associated with sc IFN β-1a tiw discontinuation at 1 year, 2 years, and 3 years are summarised in Table 2 and Additional file 1: Table S2. Two factors were identified that were independently associated with discontinuation irrespective of time on therapy. The first was increasing number of magnetic resonance imaging (MRI) scans (per one additional scan), with adjusted OR (AORs) at 1, 2 and 3 years of 1.45 (95% confidence interval [CI] 1.26–1.67), 1.18 (95% CI 1.06–1.32) and 1.20 (95% CI 1.07–1.34), respectively; the second was adherence <80% versus adherence ≥80%, with AORs at 1, 2 and 3 years of 180.95 (95% CI 135.84–241.03), 135.80 (95% CI 100.10–184.23), and 174.89 (95% CI 115.27–265.38), respectively.

**Table 2:** Adjusted odds ratios of factors associated with discontinuation of subcutaneous interferon β-1a, three times weekly, at 1, 2, and 3 years, respectively

Adjusted odds ratio (95% confidence interval)	sc IFN β-1a tiw		
	Discontinuation at 1 year (*n =* 3975)	Discontinuation at 2 years (*n =* 2592)	Discontinuation at 3 years (*n =* 1664)
Female sex (vs. male)	NS	NS	1.48 (0.98–2.22)
Region (vs. unknown)	NS	NS	NS
Age in years (continuous)	NS	NS	NS
Charlson comorbidity index (≥1 vs. 0)	NS	NS	NS
Relapses per year (continuous)	NS	1.60 (1.11–2.30)	2.31 (1.27–4.22)
High relapses (≥2 relapses) (vs. no)^a^	NS	NS	NS
DMD use history (vs. no)	NS	NS	NS
Months of treatment duration (continuous)	NS	NS	NS
No persistence (vs. yes)	NS	NS	NS
Adherence <80% (vs. ≥80%)	180.95 (135.84–241.03)	135.80 (100.10–184.23)	174.90 (115.27–265.38)
Health resource usage			
Hospital visits (1 vs. 0, 2 vs. 0, and ≥3vs. 0)	NS	NS	NS
Emergency room visits 1 vs. 0, 2 vs. 0, and ≥3vs. 0)	NS	NS	NS
Nurse visits (1 vs. 0, 2 vs. 0, and ≥3vs. 0)	NS	NS	NS
Neurologist visits (vs. 10+)			
1	0.84 (0.57–1.25)	NS	NS
2	0.67 (0.47–0.97)	NS	NS
3+	1.11 (0.84–1.48)	NS	NS
Psychologist visits (1 vs. 0, 2 vs. 0, and ≥3vs. 0)	NS	NS	NS
Psychiatrist visits (1 vs. 0, 2 vs. 0, and ≥3vs. 0)	NS	NS	NS
Speech Therapy visits (1 vs. 0, 2 vs. 0, and ≥3vs. 0)	NS	NS	NS
Outpatients (1 vs. 0, 2 vs. 0, and ≥3vs. 0)	NS	NS	NS
Increasing number of MRI scans (one additional scan versus no increase in number of MRI scans)	1.45 (1.26–1.67)	1.18 (1.06–1.32)	1.20 (1.07–1.34)
Laboratory investigations (vs. 0)			
1	0.61 (0.30–1.25)	NS	NS
2	0.93 (0.38–2.26)	NS	NS
3+	2.54 (1.20–5.38)	NS	NS
Baseline corticosteroid use (No = 0, Yes = 1)	NS	NS	NS
Follow-up			
NSAID use (vs. no)	NS	NS	NS
Antidepressants use (vs. no)	NS	1.46 (1.10–1.94)	NS
Anxiolitics use (vs. no)	1.40 (1.06–1.82)	NS	NS
Corticosteroid use (vs. no)	NS	NS	NS

*DMD* disease-modifying drug, *IFN* interferon, *MRI* magnetic resonance imaging, *NS* no significant association with discontinuation, *NSAID* non-steroidal anti-inflammatory drug, *sc* subcutaneous, *tiw* three times a week

^a^High relapse activity defined as having ≥2 relapses in the first year prior to start of subcutaneous interferon β-1a, three times weekly

Factors associated only with early discontinuation (at 1 year) were three or more sets of laboratory investigations versus no laboratory investigations (AOR 2.54, 95% CI 1.20–5.38) and anxiolytic use at follow-up (AOR 1.40, 95% CI 1.06–1.82). Factors associated only with later discontinuation (at 2 years and/or at 3 years) were antidepressant use at follow up versus no antidepressant use (AOR at 2 years 1.46, 95% CI 1.10–1.94) and a greater number of relapses (AOR at 2 years 1.60, 95% CI 1.11–2.30; AOR at 3 years 2.31, 95% CI 1.27–4.22). Results from multivariable regression when adherence was removed from the model are shown in Additional file 1: Table S3.

**Fig. 2:**
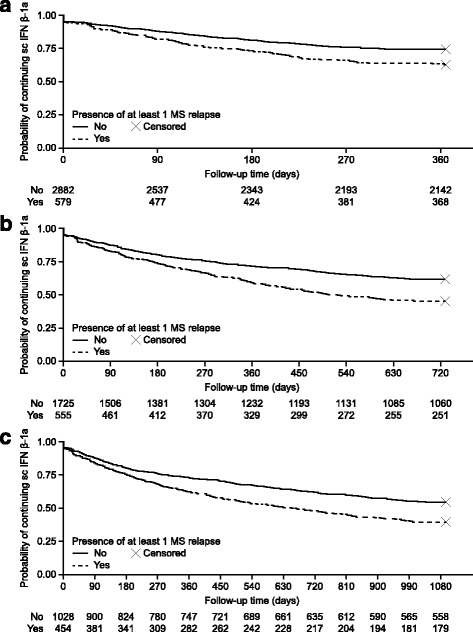
Kaplan-Meier curves of probability of sc IFN β-1a tiw continuation by follow-up time in days. **a** Data are truncated at 1 year from the index date. Kaplan-Meier curves are stratified by the presence of one or more relapses (red line) or no relapses (blue line). 3975 patients were included in the analysis; 3461 patients had two or more sc IFN β-1a tiw claims during this period. **b** Data are truncated at 2 years from the index date. Kaplan-Meier curves are stratified by the presence of one or more relapses (red line) or no relapses (blue line). 2592 patients were included in the analysis; 2280 patients had two or more sc IFN β-1a tiw claims during this period. **c** Data are truncated at 3 years from the index date. Kaplan-Meier curves are stratified by the presence of one or more relapses (red line) or no relapses (blue line). 1664 patients were included in the analysis; 1482 patients had two or more sc IFN β-1a tiw claims during this period

At 1, 2, and 3 years, patients who had ≥1 relapse were more likely to discontinue sc IFN β-1a tiw than those who had no relapses (Fig. 2a–c); at each time point, those who discontinued had a high mean number of relapses per year (≥2 relapses) in the year prior to starting treatment with sc IFN β-1a tiw than those patients who continued (Table 3).

**Table 3:** Relapse characteristics according to discontinuation status at 1, 2 and 3 years

	sc IFN β-1a tiw					
	Follow-up at 1 year		Follow-up at 2 years		Follow-up at 3 years	
	Continued	Discontinued	Continued	Discontinued	Continued	Discontinued
Total patients, *n* (%)	2505 (63.0)	1470 (37.0)	1310 (50.5)	1282 (49.5)	736 (44.2)	928 (55.8)
Relapses per year, mean (SD)	0.18 (0.52)	0.32 (0.67)	0.14 (0.34)	0.26 (0.49)	0.12 (0.26)	0.23 (0.42)
Patients with high relapses, *n* (%)^a^	97 (3.9)	84 (5.7)	46 (3.5)	73 (5.7)	21 (2.9)	52 (5.6)

*IFN*, interferon, *sc* subcutaneous, *SD* standard deviation, *tiw* three times a week

^a^High relapse activity defined as having ≥2 relapses in the first year prior to start of subcutaneous interferon β-1a, three times weekly

## Discussion

In this retrospective cohort study, discontinuation rates among US patients with MS receiving sc IFN β-1a tiw increased over time, from 36.9% at the first year to 55.8% at 3 years after treatment initiation. Poor adherence was the main factor associated with discontinuation of this therapy, irrespective of the length of time on treatment. The discontinuation rates seen in the current study are in agreement with those reported previously,[16] although other studies report a wide variation in discontinuation in patients with MS. In a respective cohort study of pharmacy claims in Germany, at 2 years post-initiation, overall persistence to one of the four first-line injected therapies was 32.3%, [17] while in a similar study approximately half of patients with MS had discontinued DMDs at the same time point [18].

Treatment adherence describes the successful self-administration of medicine by a patient, taking into account correct treatment usage with regard to administration schedule and treatment regimen (compliance) over time (persistence) [6]. Poor adherence is a common problem among patients with many types of chronic disease, including MS, and improvements in treatment adherence may have a larger effect on society and health than most therapeutic advances [2, 19]. Many factors that contribute to poor adherence to long-term therapy in patients with MS are recognised; [6] indeed, recent retrospective [7, 20] and prospective [19] observational studies indicate that the most common reasons to discontinue IFN-β therapy in real-world settings are adverse events (such as influenza-like symptoms, depression and injection-site reactions) and increased disease activity (including radiographic progression, relapses, and disability progression). Although in this study indices of disability progression were not reported, an association between relapses and discontinuation was observed at years 2 and 3, and the association with an increasing number of MRI scans may be an indicator of radiographic progression. Despite the development of strategies to help mitigate these factors, [10, 21] sustained adherence to DMDs in patients with MS remains low [16, 18].

When we compared factors associated with discontinuation in models including and excluding adherence, the only variable associated in both models was the number of laboratory investigations at 1 year (one laboratory investigation versus no laboratory investigations was inversely associated with discontinuation in the model without adherence [AOR 0.16]; three or more laboratory investigations versus no laboratory investigations was associated with discontinuation in the model that includes adherence [AOR 2.54]). For the other factors, which were significantly associated with discontinuation in only one of the models, we cannot exclude a relationship with adherence. Furthermore, the high AORs for adherence were driven by the very low proportion of patients who had adherence ≥80% but still discontinued sc IFN β-1a tiw (1 year, *n =* 68; 2 years, *n =* 66; 3 years, *n =* 41).

In this retrospective analysis, there was no significant association between age, sex, and initial DMD, respectively, and discontinuation of sc IFN β-1a tiw, which is in line with previous studies [22]. Furthermore, although overall time on treatment was not associated with discontinuation, a change was observed in the drivers of discontinuation as time on therapy increases. In the short-term (up to and including 1 year), the main drivers (an increase in laboratory investigations and an increase in the use of anxiolytics) could be associated with the common emergent adverse effects of treatment with sc IFN β-1a tiw [7]. Even though the adverse-event profile of sc IFN β-1a tiw is well-documented, consistent and stable during both clinical trials and in real-world experience, [7] adverse events can still lead to considerable discomfort and patient anxiety. Pharmacological and non-pharmacological approaches to prevent discontinuation due to adverse events have not been widely implemented in patients who discontinue sc IFN β-1a tiw therapy, resulting in missed opportunities to improve retention [16]. In the long-term (at 2 years and/or at 3 years), factors associated only with later discontinuation were antidepressant use at follow-up versus no antidepressant use and a greater number of relapses. Depression and anxiety are both comorbid conditions in patients with MS, [23] and the presence of both can contribute to poor adherence to DMDs [24]. Indeed, the 12-month prevalence of depression in patients with MS has been reported as 25.7%, and estimates of lifetime prevalence of depression are as high as 50% [25]. MS patients with comorbid depression are about half as likely to be adherent to a DMD than MS patients without depression [26]. In addition, in a real-world US health insurance-claims-based study of more than 8000 patients, depression was also a recognised adverse effect of treatment with IFN therapy (incidence rate 7.75 [95% CI 7.32–8.20]) [7]. As the current analysis shows, further investigation is required to corroborate the validity of new prescriptions of antidepressants as a driver of discontinuation at and beyond 2 years, or whether this is associated with a decrease in quality of life as a consequence of increased disease activity.

The relation between discontinuation and relapse is not straightforward. The association between the increase in the number of relapses and later discontinuation (Table 3) could be the result of the absence of perceived benefit of long-term treatment, leading to poor adherence; however, any theories on the precise nature of this association require further investigation. Although the exact causes of increased disease activity are not investigated in this retrospective real-world cohort study, there are several other possible reasons that could be investigated further, including the development of neutralising antibodies, which were shown in a European prospective multicentre centre study to develop in almost a quarter of patients on any IFN β-based regimen at a median of 23.8 months on treatment. The development of neutralising antibodies may abrogate treatment effectiveness, leading to clinical and radiological disease progression [27].

The number of patients who discontinued and switched to a different therapy increased over each of the time periods studied. Although data on the drugs to which patients switched was not analysed, the period of analysis overlaps with the date from which the oral MS drug dimethyl fumarate first became available in the USA (March 28, 2013) [28]. This also corresponds to an increase in market-based reports of dimethyl fumarate use in the final quarter of 2013 [29]. It is possible that, in response to the momentum of pre-marketing demand for an oral therapy, data collected during this study period may have included patients who switched from injected to oral therapies. Additionally, the current study showed that patients who had a history of using other DMDs may be more likely to switch to another medication after discontinuing sc IFN β-10 tiw. However, this period of overlap (9 months) is too short to assess the impact on discontinuation of sc IFN β-1a tiw.

One limitation of this retrospective study is that, as an analysis of administrative health care claims data, it does not take into account all clinical information (such as MS subtype and disease severity), socioeconomic status, enrolment in patient-support programmes, and other factors that might influence discontinuation. However, claims database analyses may have an advantage over retrospective chart review for identifying the causes for discontinuation over time, as they provide a precise record of the duration of treatment in the broader patient population and do not have the biases associated with reporting in clinical trials or post-marketing observational studies [7].

## Conclusion

In conclusion, increased awareness among physicians of the clinical significance of the length of time on treatment could foster a culture where patients are actively asked by physicians whether they are experiencing any time-specific adverse events, rather than reliance on emergent or retrospective reporting by patients. Such improved awareness could lead to earlier access to disease-management strategies and patient-support services and could inform proactive preventive treatment strategies to improve long-term treatment adherence in patients with MS.

## Additional file

Additional file 1: Table S1. Health care resource usage of patients with multiple sclerosis initiating subcutaneous interferon β-1a, three times weekly by discontinuation status. **Table S2.** Crude odds ratios of factors associated with discontinuation of subcutaneous interferon β-1a, three times weekly, at 1, 2, and 3 years, respectively. **Table S3.** Adjusted odds ratios of factors associated with discontinuation of subcutaneous interferon β-1a, three times weekly, at 1, 2, and 3 years, respectively, with adherence removed from the model. (DOCX 36 kb)
