# Supplementary material for: Reasons for discontinuation of subcutaneous interferon β-1a three times a week among patients with multiple sclerosis: a real-world cohort study
Source: BMC Neurol. 2017 Mar 23;17:57. doi: 10.1186/s12883-017-0831-4 (PMC5364602; doi:10.1186/s12883-017-0831-4)
Supplement: Additional file 1: Table S1. — Health care resource usage of patients with multiple sclerosis initiating subcutaneous interferon β-1a, three times weekly by discontinuation status. Table S2. Crude odds ratios of factors associated with discontinuation of subcutaneous interferon β-1a, three times weekly, at 1, 2, and 3 years, respectively. Table S3. Adjusted odds ratios of factors associated with discontinuation of subcutaneous interferon β-1a, three times weekly, at 1, 2, and 3 years, respectively, with adherence removed from the model. (DOCX 36 kb) [file 12883_2017_831_MOESM1_ESM.docx]

**Supplementary Table 1. Health care resource usage of patients with multiple sclerosis initiating subcutaneous interferon β-1a, three times weekly by discontinuation status**

| **Patients, n (%)** | **sc IFN β-1a tiw** | |
| --- | --- | --- |
|  | **All patients (N=5956)** | **Discontinued patients (N=2862)** |
| Number of hospital admissions |  |  |
| 0 | 5108 (85.8) | 2273 (79.4) |
| 1 | 506 (8.5) | 330 (11.5) |
| 2 | 174 (2.9) | 124 (4.3) |
| 3+ | 168 (2.8) | 135 (4.7) |
| Number of emergency room visits |  |  |
| 0 | 5663 (95.1) | 2646 (92.5) |
| 1 | 202 (3.4) | 141 (4.9) |
| 2 | 46 (0.8) | 37 (1.3) |
| 3+ | 45 (0.8) | 38 (1.3) |
| Number of nurse visits |  |  |
| 0 | 5281 (88.7) | 2426 (84.8) |
| 1 | 319 (5.4) | 200 (7.0) |
| 2 | 132 (2.2) | 87 (3.0) |
| 3+ | 224 (3.8) | 149 (5.2) |
| Number of neurologist visits |  |  |
| 0 | 2155 (36.2) | 752 (26.3) |
| 1 | 387 (6.5) | 181 (6.3) |
| 2 | 434 (7.3) | 182 (6.4) |
| 3+ | 2980 (50.0) | 1747 (61.0) |
| Number of psychologist visits |  |  |
| 0 | 5561 (93.4) | 2608 (91.1) |
| 1 | 125 (2.1) | 83 (2.9) |
| 2 | 65 (1.1) | 45 (1.6) |
| 3+ | 205 (3.4) | 126 (4.4) |
| Number of psychiatrist visits |  |  |
| 0 | 5511 (92.5) | 2558 (89.4) |
| 1 | 100 (1.7) | 73 (2.6) |
| 2 | 49 (0.8) | 25 (0.9) |
| 3+ | 296 (5.0) | 206 (7.2) |
| Number of speech therapy visits |  |  |
| 0 | 5827 (97.8) | 2773 (96.9) |
| 1 | 66 (1.1) | 48 (1.7) |
| 2 | 24 (0.4) | 20 (0.7) |
| 3+ | 39 (0.7) | 22 (0.8) |
| Number of outpatient visits |  |  |
| 0 | 583 (9.8) | 153 (5.3) |
| 1 | 189 (3.2) | 36 (1.3) |
| 2 | 158 (2.7) | 36 (1.3) |
| 3+ | 5026 (84.4) | 2637 (92.1) |
| Number of MRI scans |  |  |
| 0 | 1999 (33.6) | 626 (21.9) |
| 1 | 1459 (24.5) | 640 (22.4) |
| 2 | 1006 (16.9) | 550 (19.2) |
| 3+ | 1492 (25.1) | 1046 (36.5) |
| Number of laboratory investigations |  |  |
| 0 | 5424 (91.1) | 2584 (90.3) |
| 1 | 164 (2.8) | 78 (2.7) |
| 2 | 100 (1.7) | 50 (1.7) |
| 3+ | 268 (4.5) | 150 (5.2) |

DMD, disease-modifying drug; IFN, interferon; MRI, magnetic resonance imaging; NSAID, non-steroidal anti-inflammatory drugs; sc, subcutaneous; tiw, three times a week.

**Supplementary Table 2. Crude odds ratios of factors associated with discontinuation of subcutaneous interferon β-1a, three times weekly, at 1, 2, and 3 years, respectively**

| **Crude odds ratio**  **(95% confidence interval)** | **sc IFN β-1a tiw** | | |
| --- | --- | --- | --- |
|  | **Discontinuation at 1 year (n=3975)** | **Discontinuation at 2 years (n=2592)** | **Discontinuation at 3 years (n=1664)** |
| Female sex (vs. male) | 1.25 (1.08–1.46) | 1.20 (0.99–1.43) | 1.32 (1.05–1.65) |
| Region (vs. unknown) |  |  |  |
| Northeast | 0.81 (0.41–1.59) | 1.46 (0.47–4.52) | 2.23 (0.40–12.40) |
| North Central | 0.69 (0.35–1.34) | 1.26 (0.41–3.89) | 1.96 (0.36–10.80) |
| South | 0.98 (0.50–1.91) | 1.72 (0.56–5.31) | 2.90 (0.53–15.98) |
| West | 1.00 (0.51–1.96) | 1.97 (0.64–6.11) | 3.24 (0.59–17.96) |
| Age in years (continuous) | 0.99 (0.98–0.99) | 0.99 (0.98–1.00) | 1.00 (0.99–1.00) |
| Charlson comorbidity index (vs. 0) |  |  |  |
| Index = 1 | 1.18 (0.99–1.40) | 1.08 (0.88–1.32) | 1.11 (0.86–1.44) |
| Index = 2 | 0.96 (0.76–1.21) | 1.02 (0.77–1.36) | 1.12 (0.77–1.61) |
| Index ≥ 3 | 1.28 (0.98–1.67) | 1.22 (0.87–1.72) | 1.09 (0.69–1.74) |
| Relapses per year (continuous) | 1.49 (1.33–1.67) | 2.22 (1.79–2.75) | 2.93 (2.70–4.20) |
| High relapses (≥2 relapses) (vs. no)* | 1.51 (1.12–2.03) | 1.67 (1.14–2.42) | 2.02 (1.21–3.39) |
| DMD use history (vs. no) | 1.43 (1.25–1.63) | 1.33 (1.13–1.56) | 1.28 (1.05–1.56) |
| Months of treatment duration (continuous) | 0.13 (0.10–0.15) | 0.32 (0.28–0.37) | 0.41 (0.36–0.47) |
| No persistence (vs. yes) | 1.21 (0.87–1.70) | 1.85 (1.34–2.54) | 2.01 (1.37–2.96) |
| Adherence <80% (vs. ≥80%) | 159.33  (121.31–209.27) | 133.37  (98.99–179.69) | 149.79  (102.12–219.73) |
| Health resource usage |  |  |  |
| Hospital visits (vs. 0) |  |  |  |
| 1 | 1.74 (1.36–2.22) | 1.21 (0.93–1.56) | 1.20 (0.89–1.62) |
| 2 | 1.77 (1.07–2.91) | 1.90 (1.19–3.02) | 1.76 (1.07–2.87) |
| 3+ | 4.14 (2.24–7.62) | 4.18 (2.35–7.42) | 4.54 (2.42–8.50) |
| Emergency room visits (vs. 0) |  |  |  |
| 1 | 1.87 (1.25–2.80) | 1.61 (1.05–2.47) | 1.52 (0.92–2.52) |
| 2 | 4.02 (1.65–9.80) | 3.80 (1.41–10.27) | 2.01 (0.83–4.88) |
| 3+ | 9.67 (2.14–43.68) | 1.98 (0.84–4.69) | 7.88 (1.83–33.93) |
| Nurse visits (vs. 0) |  |  |  |
| 1 | 1.01 (0.74–1.40) | 1.10 (0.80–1.51) | 0.96 (0.67–1.38) |
| 2 | 1.35 (0.81–2.24) | 1.33 (0.80–2.20) | 1.55 (0.83–2.76) |
| 3+ | 2.33 (1.50–3.60) | 1.88 (1.26–2.81) | 1.13 (0.73–1.75) |
| Neurologist visits (vs. 10+) |  |  |  |
| 1 | 0.92 (0.73–1.16) | 1.24 (0.84–1.83) | 1.09 (0.59–1.99) |
| 2 | 0.60 (0.48–0.75) | 0.66 (0.46–0.95) | 0.63 (0.37–1.05) |
| 3+ | 0.99 (0.85–1.16) | 0.82 (0.67–1.00) | 0.83 (0.64–1.08) |
| Psychologist visits (vs. 0) |  |  |  |
| 1 | 1.79 (1.09–2.95) | 1.36 (0.82–2.26) | 1.69 (0.92–3.10) |
| 2 | 1.12 (0.48–2.58) | 1.35 (0.67–2.72) | 1.19 (0.50–2.79) |
| 3+ | 1.27 (0.87–1.87) | 1.37 (0.92–2.02) | 1.37 (0.89–2.10) |
| Psychiatrist visits (vs. 0) |  |  |  |
| 1 | 2.36 (1.43–3.91) | 1.81 (0.95–3.44) | 1.18 (0.57–2.42) |
| 2 | 1.18 (0.69–2.04) | 1.97 (0.94–4.13) | 1.58 (0.63–3.98) |
| 3+ | 1.91 (1.35–2.70) | 2.05 (1.46–2.87) | 2.08 (1.43–3.02) |
| Speech Therapy visits (vs. 0) |  |  |  |
| 1 | 3.30 (1.64–6.65) | 1.81 (0.89–3.70) | 1.94 (0.92–4.08) |
| 2 | 0.86 (0.26–2.86) | 1.21 (0.41–3.61) | 2.02 (0.63–6.50) |
| 3+ | 0.86 (0.35–2.14) | 1.73 (0.75–3.96) | 1.16 (0.44–3.05) |
| Outpatients (vs. 0) |  |  |  |
| 1 | 0.54 (0.26–1.12) | NS | NS |
| 2 | 0.41 (0.21–0.82) | 0.57 (0.07–4.64) | NS |
| 3+ | 0.48 (0.29–0.79) | 0.84 (0.28–2.50) | 1.90 (0.32–11.37) |
| Increasing number of MRI scans (one additional scan versus no increase in number of MRI scans) | 1.22 (1.34–1.30) | 1.67 (1.10–1.24) | 1.78 (1.11–1.25) |
| Laboratory investigations (vs. 0) |  |  |  |
| 1 | 0.62 (0.40–0.97) | 0.67 (0.41–1.09) | 1.02 (0.54–1.90) |
| 2 | 1.11 (0.69–1.80) | 0.88 (0.49–1.58) | 1.45 (0.69–3.04) |
| 3+ | 1.43 (0.98–2.08) | 0.87 (0.61–1.24) | 0.93 (0.62–1.40) |
| Baseline corticosteroid use (No=0, Yes=1) | 1.11 (0.98–1.27) | 1.06 (0.91–1.25) | 1.02 (0.84–1.24) |
| Follow-up |  |  |  |
| NSAID use (vs. no) | 1.52 (1.33–1.73) | 1.67 (1.43–1.96) | 1.44 (1.15–1.81) |
| Antidepressants use (vs. no) | 1.47 (1.26–1.70) | 1.58 (1.32–1.88) | 1.80 (1.48–2.20) |
| Anxiolitics use (vs. no) | 1.36 (1.19–1.55) | 1.50 (1.28–1.75) | 1.70 (1.37–2.12) |
| Corticosteroid use (vs. no) | 1.25 (1.08–1.465) | 1.20 (1.00–1.43) | 1.44 (1.18–1.75) |

DMD, disease-modifying drug; IFN, interferon; MRI, magnetic resonance imaging; NS, no significant association with discontinuation; NSAID, non-steroidal anti-inflammatory drug; sc, subcutaneous; tiw, three times a week.

*High relapse activity defined as having ≥2 relapses in the first year prior to start of subcutaneous interferon β-1a, three times weekly

**Supplementary Table 3. Adjusted odds ratios of factors associated with discontinuation of subcutaneous interferon β-1a, three times weekly, at 1, 2, and 3 years, respectively, with adherence removed from the model**

| **Adjusted odds ratio**  **(95% confidence interval)** | **sc IFN β-1a tiw** | | |
| --- | --- | --- | --- |
|  | **Discontinuation at 1 year (n=3975)** | **Discontinuation at 2 years (n=2592)** | **Discontinuation at 3 years (n=1664)** |
| Female sex (vs. male) | NS | NS | NS |
| Region (vs. unknown) | NS | NS | NS |
| Age in years (continuous) | 0.96 (0.93-0.99) | NS | NS |
| Charlson comorbidity index (≥1 vs. 0) | NS | NS | NS |
| Relapses per year (continuous) | NS | NS | NS |
| High relapses (≥2 relapses) (vs. no)* | NS | NS | NS |
| DMD use history (vs. no) | NS | NS | NS |
| Months of treatment duration (continuous) | 0.21 (0.16-0.26) | 0.21 (0.15-0.31) | 0.26 (0.17-0.39) |
| No persistence (vs. yes) | NS | NS | NS |
| Health resource usage |  |  |  |
| Hospital visits (vs. 0) | NS | NS | NS |
| 1 | 0.37 (0.15-0.94) | NS | NS |
| 2 | 0.21 (0.04-0.96) | NS | NS |
| 3+ | 0.67 (0.20-2.27) | NS | NS |
| Emergency room visits (≥1 vs. 0) | NS | NS | NS |
| Nurse visits (≥1 vs. 0) | NS | NS | NS |
| Neurologist visits (vs. 10+) |  |  |  |
| 1 | NS | NS | NS |
| 2 | NS | NS | NS |
| 3+ | NS | NS | NS |
| Psychologist visits (≥1 vs. 0) | NS | NS | NS |
| Psychiatrist visits (≥1 vs. 0) | NS | NS | NS |
| Speech Therapy visits (≥1 vs. 0) | NS | NS | NS |
| Outpatients (≥1 vs. 0) | NS | NS | NS |
| Increasing number of MRI scans (one additional scan versus no increase in number of MRI scans) | NS | NS | NS |
| Laboratory investigations (vs. 0) |  |  |  |
| 1 | 0.16 (0.04–0.67) | NS | NS |
| 2 | 8.68 (0.60–124-75) | NS | NS |
| 3+ | 2.13 (0.59–7.66) | NS | NS |
| Baseline corticosteroid use (No=0, Yes=1) | NS | NS | NS |
| Follow-up |  |  |  |
| NSAID use (vs. no) | NS | NS | NS |
| Antidepressant use (vs. no) | 1.89 (1.04-3.41) | NS | 4.20 (1.00-17.69) |
| Anxiolytic use (vs. no) | NS | NS | NS |
| Corticosteroid use (vs. no) | NS | NS | NS |

DMD, disease-modifying drug; IFN, interferon; MRI, magnetic resonance imaging; NS, no significant association with discontinuation; NSAID, non-steroidal anti-inflammatory drug; sc, subcutaneous; tiw, three times a week.

*High relapse activity defined as having ≥2 relapses in the first year prior to start of subcutaneous interferon β-1a, three times weekly
